# A robust intelligent zero-day cyber-attack detection technique

**DOI:** 10.1007/s40747-021-00396-9

**Published:** 2021-05-28

**Authors:** Vikash Kumar, Ditipriya Sinha

**Affiliations:** Department of Computer Science and Engineering, National Institute of Technology Patna, Patna, 800005 India

**Keywords:** Cyber-attacks, Zero-day attack, Heavy-hitters, Signature generation, Token extraction, High volume attack, Low volume attack

## Abstract

With the introduction of the Internet to the mainstream like e-commerce, online banking, health system and other day-to-day essentials, risk of being exposed to various are increasing exponentially. Zero-day attack(s) targeting unknown vulnerabilities of a software or system opens up further research direction in the field of cyber-attacks. Existing approaches either uses ML/DNN or anomaly-based approach to protect against these attacks. Detecting zero-day attacks through these techniques miss several parameters like frequency of particular byte streams in network traffic and their correlation. Covering attacks that produce lower traffic is difficult through neural network models because it requires higher traffic for correct prediction. This paper proposes a novel robust and intelligent cyber-attack detection model to cover the issues mentioned above using the concept of heavy-hitter and graph technique to detect zero-day attacks. The proposed work consists of two phases (*a*) Signature generation and (*b*) Evaluation phase. This model evaluates the performance using generated signatures at the training phase. The result analysis of the proposed zero-day attack detection shows higher performance for accuracy of 91.33% for the binary classification and accuracy of 90.35% for multi-class classification on real-time attack data. The performance against benchmark data set CICIDS18 shows a promising result of 91.62% for binary-class classification on this model. Thus, the proposed approach shows an encouraging result to detect zero-day attacks.

## Introduction

The digitization of service and other activities turned the Internet into an inevitable part in various tasks. It makes a more significant proportion of the population dependent on the Internet for their daily activities (e.g., gaming, shopping, chatting, financial activities, study, etc.), making them prone to several threats and attacks. A person sitting at one end can easily access others’ information at different ends within a fraction of a second due to the Internet’s globalization. Detecting malicious activities and offering a secure environment against the Internet’s sophisticated traffic of a diverse set of users are the top priorities of security firms.

**Fig. 1:**
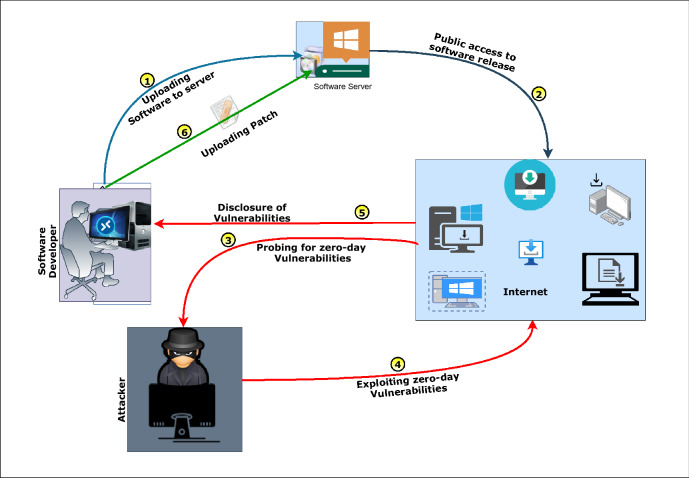
Different phases of ZA

Today, the world is going through a COVID-19 pandemic. Attackers are looking for every possible way to execute their malicious intent. As per the report published in [1], during the COVID-19 crisis, attackers targeted consumers and enterprises through a themed attack. Reports from [2] show the rise in different types of cyber threats during the COVID-19 pandemic. The phishing attack variants have the highest occurrence followed by malware/ ransomware attacks. The cost of ransomware is spiked to US$ 20 Billion against US$ 11.5 Billion in 2019 [3]. According to the Cisco reported by cyber defense magazine, [3] the trend in the growth of ransomware attack is 350% annually and the expected expenditures on cyber-security are to reach $1 trillion by 2024. Providing security to a network or organization is becoming arduous with time due to the increasing traffic complexity. For the real-time environment, protecting against threats is a sufficient task and minimizing the false alarm rate is another inevitable part of the cyber defense mechanism. According to IBM [3], only 38% of global organizations claim that they can handle sophisticated cyber-attacks. Many approaches [4–6] work efficiently for the attacks whose signatures are available publicly to the security experts. Along with that, an appreciable amount of researches are ongoing to defend and mitigate unknown threats or zero-day attacks (ZAs). Here, ZA refers to those malicious activities that involve exploiting an unknown vulnerability of a system or software. In [7], authors reviewed existing works, mainly aiming to detect clone attacks performed through clone nodes. All these attack detection schemes are analyzed and compared for static and dynamic wireless sensor networks (WSNs). The vulnerabilities in zero-day threats are only known to the black-hat community. They exploit them until the vendor provides a patch to install on all the systems. Figure 1 explains the different phases of the ZA scenario. Here the developers first release software with overlooked glitches/ vulnerabilities. The Black-hat community discovers those vulnerabilities or glitches present in the software and then performs a zero-day exploit against those vulnerabilities. Once the developers become aware of any such exploits, they develop a patch for those vulnerabilities. They release a patch to all users to avoid further attack possibilities due to that particular glitch.

*Motivation:* Recently, in 2020, Microsoft has faced ZA [8] caused by the Adobe Type Manager (ATM) library. This attack targeted the remote code execution vulnerabilities in ATM. It gives attackers to run malicious scripts remotely that are sent through spam or downloaded unknowingly. The ATM mentioned above vulnerability mentioned above could lead to a ransomware attack by executing some malicious code. Another one is the CVE-2020-0674 [9] vulnerability, whose source is the Internet Explorer scripting engine. The attack due to this vulnerability affected IE v9-11 through phishing emails or link redirection. The other ZA, whose victim itself is a security software firm Sophos. The attack executed against Sophos XG firewall due to the CVE-2020-12271 [10] vulnerability. This attack can change firewall settings, grant unauthorized access to a system, or uses malware installation. There are many more ZAs that are being performed and still unobserved. According to report [1] during the covid period, different types of unknown cyber-attacks are increasing rapidly. It motivates authors to design a novel technique to prevent zero-day cyber-attacks.

This paper proposes a framework to detect unknown cyber-attacks (ZAs) by introducing a robust intelligent, novel approach that combines the concept of heavy-hitters (HH) and graph technique. This model covers unknown high volume attacks (HVA), i.e., variants of DoS/DDoS attacks and unknown low volume attacks (LVA), i.e., variants of data-theft attack, scanning, etc. Signatures or patterns of those attacks are unknown to the vendors. Based on study [11] this paper concludes that it is complicated and challenging to ensure that an attack comprises a sequence of all unknown exploits [11]. A ZA usually consists of both known and unknown exploits [11]. Hence, the ZA detection can be achieved through known exploit signatures discovered in any traffic.

*Contribution:* Contributions of the proposed work can be summarized as follows-

1. An integration of HH and graph-based technique is proposed to design a robust intelligent system that enables on-the-fly detection of ZAs.
2. The proposed system can cover the detection of broader categories of ZAs by utilizing up-to-date network traffic of known attacks.
3. The model is designed based on a raw byte stream of captured real-time network traffic.
4. The proposed model is independent of network, source and destination-specific information.
5. Performance of proposed model compared to existing approaches for both binary and multi-class classification show better result.
6. The proposed system evaluates ZAs detection’s performance based on the real-time traffic logged by the network and the latest benchmark data set CICIDS18.

*Paper Organization:* The paper is organized in the following sections as Sect. 2 deals with the preliminary concepts required to design the proposed work. It reviews in brief recent contributions in the field of ZA detection Sect. 3. Section 4 discusses other threat models and solutions by the proposed work. Section 5 gives a detailed analysis of the proposed work. Section 6 provides details of the experimental setup to generate test data for the proposed work is discussed. Section 7 explains the different performance metrics and result analysis, followed by Sect. 8 which concludes the piece by highlighting the advantages and limitations of the proposed framework.

## Preliminaries

Two preliminary techniques applied to detect zero-day threats in our proposed approach are (*A*) HH problem and (*B*) Graph-based approach.

Explanation the above two approaches are as follow:

*A.*: *HH problem* It is a frequency estimation problem for a stream of data where the goal is to find tokens out of input data stream $\sigma$ that qualify the cut-off frequency. Here, the token refers to fixed length (say k) sequences of characters for a given string $\sigma$. Let’s assume, $\mathbf{S}=\{s_{1}s_{2}s_{3}...s_{i}\}$ is a set of tokens obtained from $\sigma$ consisting with *u* unique elements.If initially, total of *N* tokens are present in the input stream not necessarily distinct with each $i^{th}$ token in *S* having a frequency $f_{i}$, then we say,
  $$\begin{aligned} f_{1}+f_{2}+...+f_{l}=N \end{aligned}$$
  Several algorithms exist for frequency estimation problems [12–15]. Metwally et al. [13] are one of them. Algorithm 1 describes the modified version of the algorithm mentioned above. This algorithm estimates the frequency of tokens in the proposed approach. It is a space-saving algorithm and provides a more accurate estimator than other existing methods [12, 14, 15]. It returns the top pre-specified number (**z**) of frequent tokens. *freq*[], $cutoff\_freq$ and *y* are the list of frequent tokens, minimum cutoff on the number of occurrences of each token and sliding window size respectively.

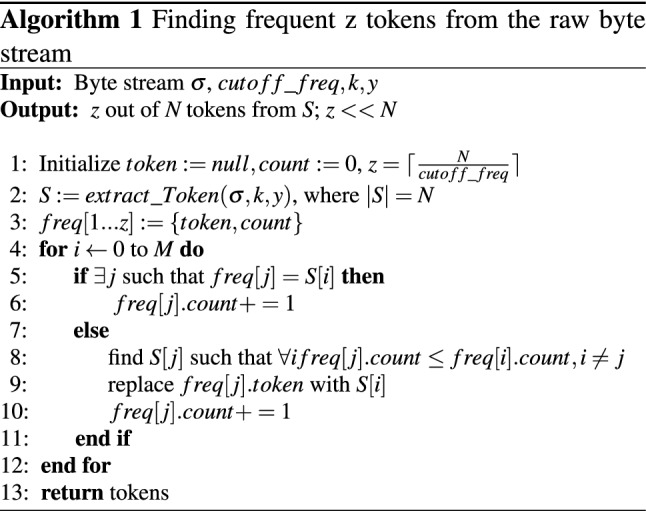

*B.*: *Graph* A graph G(V,E) is a set of vertices V and edges E where the members of set E represent the interconnection between set V nodes.

### Definition 1

* Token Graph* *G*(*I*, *E*) :  Tokens set *I* is a variable-length unique sequence of hexadecimal characters . A token graph is a graph whose vertices are a set of tokens and the connectivity among those vertices is shown by directed edges $e_{i}\in E$.

### Definition 2

**Fig. 2:**
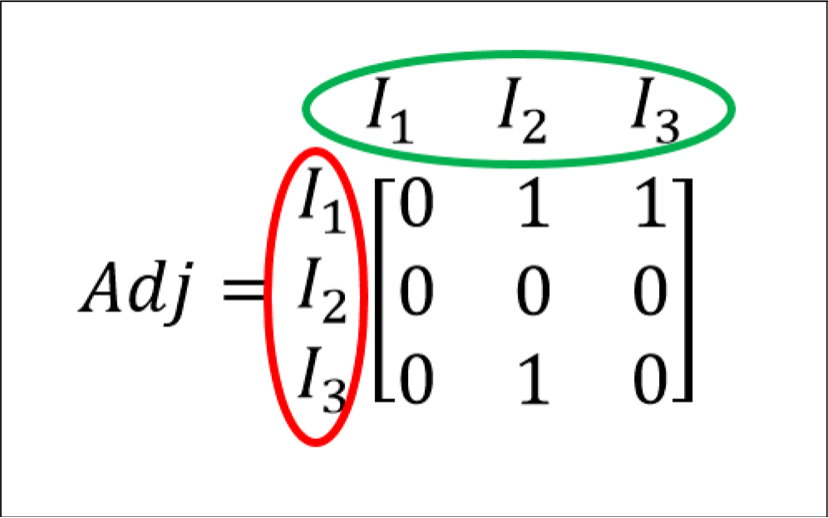
Example of the adjacency matrix for the above connectivity

*Adjacency Matrix (Adj):* For the token graph, it is a two-dimensional matrix. Figure 2 shows the ‘to’and‘from’ relation using green and red encircled vertices respectively for dependency in a graph with an edge “$from\rightarrow to$”. The value 1 and 0 indicate the presence and absence of a directed edge between vertices, respectively. Let’s assume, $I_{1}\rightarrow I_{2},I_{1}\rightarrow I_{3},I_{3}\rightarrow I_{2}$, are three connectivity present in an token graph. Figure 2 shows the adjacency matrix for the presence of connections.

### Definition 3

*Vertex score function:* A function $f_{x}$ is assigned a score value to each vertex or tokens in an token graph and is defined by Eq. 1

1 $$\begin{aligned} f_{x}(x,d_{1},d_{2})=\frac{\mathrm{estimated}\ \mathrm{counter} \ \mathrm{of}\ x\ \mathrm{in}\ d_{1}}{\mathrm{estimated}\ \mathrm{counter}\ \mathrm{of}\ x\ \mathrm{in}\ d_{2}+1} \end{aligned}$$

Where *x* is a particular token,

$$d_{1},d_{2} $$ are the set of different input stream files.

## Related work

Detection of ZA has recently gained tremendous attention. Several solutions exist, ranging from behavioral-based to graph-based approaches from packet-level to kernel-level. Table 1 shows the summary of different ZAs detection approaches.

***Anomaly-based***

An anomaly detection approach in [16] is presented using logistic regression for chaotic in-variants, i.e., correlation dimensions, entropy, etc., which are intrinsically non-linear features. According to the authors, these properties produce highly discriminating attributes that machine learning algorithms can use. The proposed scheme is not suitable for anomalous attacks that target packets’ content, buffer overflow or attacks associated with exploiting vulnerabilities. In [17], Duessel et al. have proposed a ZA detection technique at the application layer by presenting a new data representation called $c_{n}-$gram. It allows the fusion of syntactic and sequential attributes of the packet payloads in combined feature space. The similarity of mapped byte messages combined with syntax-level attributes using the data representation is calculated after the detection algorithm’s training to learn the global normality. Detection is done by comparing the learned model’s message and assigning a score for the extent of anomalous behavior. In [18], Moon et al. have proposed a host-based detection system for secure human-centric computation. To detect whether a process executes on the host PC is malicious or not, they define 39 features under seven categories (i.e., process, thread, file system, registry, etc.). They also create a database for these features collected from the host PC. These features are mapped to a feature vector used by the decision tree to classify malware and benign programs.

In [19], Moustafa et al. have proposed an Outlier Dirichlet Mixture (ODM) based detection system for fog. In [20], authors have proposed an architecture to detect zero-day polymorphic worms attack using signature, behavior, and anomaly-based technique. The proposed architecture consists of three layers, namely: detection, analysis and resource layer. The detection engine uses good traffic and malicious traffic and for ZA detection. In [21], Khan et al. have proposed a multilevel anomaly detection for supervisory control and data acquisition systems (SCADA). Their model is based on the expected and consistent communication structure that takes place among devices in setup. To build the model, they have preprocessed the data applying dimensionality reduction technique and then create the signature database using Bloom filter. Contents-level detection is integrated with an instance-based learner to make the model hybrid ZAs detection.

The above state-of-the-arts focused on generating discriminating features, new data representation to combine the byte-level information with syntax-level information and the consistent behavior analysis for anomaly detection. Hence, somehow these techniques rely on the normal behavior of network traffic to detect any ZA activity.

***Graph-based***

Attack detection through graphical models has shown a significant improvement over the behavioral-based (or, anomaly-based) attack detection. Recent works [11, 22–26] have used different concepts to implement graphical models. In [27], authors have proposed an anomaly detector using the likelihood ratio of network attacks. They treat a computer network as a directed graph where a node refers to hosts and the edge between them represents communication taking place. They first introduce a stochastic attacker behavior model and then use the detector to compare network behavior probability when the attacker compromises the hosts under the normal condition. In [23], Wang et al. have proposed the DaMask architecture to detect the variants of DDoS attack, which uses Bayesian network inference in which the model gets auto-update according to new observations. In [25], Singh et al. have proposed a layered architecture for ZA detection using an attack graph. The layers of the architecture are the ZA path generator, risk analyzer, and physical layer. This architecture of a centralized database and server used for other layers. They have proposed an algorithm called “AttackRank” to find the likelihood of exploits in the graph. In [26], Yichao et al. have proposed a solution that discovers the effective attack path through compact graph planning. The solution consists of three steps which are formalism and closure calculation, graph construction and finally, the attack path extraction. In [22], Bayoglu et al. have proposed a content-based graphical framework to classify polymorphic worm’s signature by Conjunction of Combinational Motifs (CCM). Invariant parts of worms are used as vertices of the graph. This CCM automatically generates signatures for unseen polymorphic worms and detects them.

In [11], Sun et al. have proposed a graph-based technique ZePro, to identify the ZA path. This technique uses the Bayesian network to assign probabilities to each vertex based on the intrusion evidence. The proposed solution is implemented at the kernel level using the object instances as vertex and is the modification of the Patrol technique [28]. The system’s accuracy towards the finding of a ZA path depends on the evidence provided. In [24], AlEroud et al. have proposed an approach to detect cyber-attacks on software-defined networks. This approach is based on the inference mechanism to reduces false predictions. To detect ZAs, a run-time graph is created based on the similarity of the network flow based on labels (i.e., target class). The node represents the type of alerts or benign activity and the relationship between them is the similarity of the features of nodes. Those alerts are generated using rules accessed and updated by the network administrator. A graph is used to retrieve related nodes that produce a higher rate of attack detection. In [29], authors have proposed an approach to detect botnet in the IoT environment which is based on extracting high-level features through function-call graphs. Their approach consists of four steps which are- (*a*) Generating function-calls (*b*) Generating PSI-Graph (*c*) Preprocessing and (*d*)Classification. Here, processing and converting the PSI-Graph into a numeric vector and then a CNN is used to classify it into two classes $non-attack$ and *botnet*.

The basic building block of the frameworks reviewed in this subsection is the graph where nodes and edges are treated according to the implementation. E.g., few works treated hosts as nodes and communication between nodes as edges. On the other hand, some treat nodes as an instance of file structures and edges as an ordinal relationship. A few of them are based on external evidence that assigns a likelihood of a node as malicious. In contrast, others give the likelihood by comparing network behavior under normal conditions and attack conditions. Overall, these approaches extract the signature through the graph by applying specific criteria to detect ZAs.

***ML and deep learning based***

This section describes the approaches that apply ML and DNN techniques to propose their framework.

Tran et al. [30] have proposed a Cyber Resilience Recovery Model (CRRM) which handles the outbreaks in closed networks. The NIST SP 800-61 incident response framework for standard and resilience is integrated with Susceptible-Infected-Quarantined-Recovered (SIQR) model [31] to capture ZAs and recovery. In [32], authors have proposed a detection technique using a fog ecosystem for the Internet of Things (IoT) environment using a deep learning approach. Due to the fog network’s closeness to the smart infrastructures, fog nodes are accountable for training the models and performing attack detection. The training model results in attack detection models and associated native learning parameters used by fog nodes for global update and propagation. In [33], Saied et al. have proposed a detection approach using Artificial Neural Network (ANN) for known and unknown DDoS attacks based on specific features that can distinguish DDoS from genuine traffic. The model is trained using Java Neural Network Simulator (JNNS) on preprocessed data and integrated with Snort-AI. A Gated Recurrent Unit (GRU) based approach is proposed in [34]. The main objective of the work is to detect new DDoS attacks. As per the claim made by the authors, the proposed model shows higher accuracy. CANintelliIDS [35], an approach proposed to mitigate the security issue of the in-vehicle communication that are prone to various attacks. It combines concurrent neural network (CNN) and GRU techniques to detect possible attacks.

In [36], Afek et al. have proposed a signature extraction technique for high volume ZAs by using the concept of heavy-hitters. They have particularly followed Metwally’s heavy-hitter algorithm with slight modifications and generated all possible sets of k-grams from the input data. The idea behind detecting zero-day DDoS attacks is to find heavy-hitters in attack data and genuine data. Now, heavy hitters are compared in each data and placed in a different category based on their extent of being malicious based on a predefined threshold. Finally, by filtering out the most probable malicious heavy-hitters, they can find out the attacks.

In [37], Kim et al. have proposed a malware detection system using deep learning called “transferred deep- convolution generative adversarial networks (tDCGAN)”. The deep auto-encoder is used to learn the malware characteristics and used decoder to produce new data. Then transfer these to the adversarial network generator. The proposed system has three parts: compression and reconstruction of data, generating fake malware data, and finally detecting malware. In [38] authors have proposed a DNN based approach to detect cyber-attacks. It uses a hybrid technique using PCA and GWO algorithm where first PCA reduces the dimension of the data set and GWO is used to optimize the transformed data set to reduce the redundancy in the transformed data set. This approach mainly focuses on reducing the dimensionality to make DNN-based IDS detection more responsive.

In [39], authors have proposed a framework that can efficiently handle the volatility of historical attack data and also the multivariate dependency among the attacks. The main objective is to disclose the dependence and trend among various vulnerabilities and exploits on volatile historical data using copula. Gaussian and Student-t are used as the copula function. This function gives the joint property of attack risks. In [40], authors propose an anomaly detection approach using a multi-stage attention mechanism along with LSTM based CNN model. The proposed method specifically covers the abnormality in data generated through various sensors in automated vehicles. They also proposed an ensemble approach that uses a voting technique to decide on anomalous data from different classifiers.

In [41], authors addressed the issue with detecting ZAs due to the lack of labeled attack data. They use a manifold alignment approach that maps the source and target data domain into the same latent space. It assists in getting rid of the different feature spaces and probability distribution within domains. The generated space is also subject to a newly proposed technique that produces soft labels to cope with the lack of labels used to create the DNN model for ZA detection. In [42], authors have proposed an intrusion detection and prevention system for the cloud using classification and one-time signature (OTS) technique. The OTS is used to access the data on the cloud, which is different from one-time password OTP. They have used hybrid classification by combining normalized k-means with the recurrent neural network. In [43], authors have proposed an autoencoder-based deep approach for ZA detection. The authors demonstrate the performance of the proposed method using two well-known data sets, NSL-KDD and CICIDS2017. The performance is compared with the one-class SVM outlier detection.

Most of the work reviewed in this subsection applies a deep learning approach and very few worked with classical ML techniques. The advantage of the deep learning technique is that it requires minimal or no feature engineering and learns the distribution of data sets. The disadvantage of deep learning is that it needs massive data samples to learn the prediction model correctly. Hence, these approaches overlook attack categories that generate low traffic or whose samples are meager in number.

*Limitations of State-of-arts:* Review of state-of-the-arts conclude that the existing works apply ML/DNN technique, graph and anomaly-based approaches for ZAs detection. Most of them are only considering HVA detection. Though zero-day LVAs are harmful for a system or organization, the existing methods overlooked them by focusing on DoS/DDoS attacks’ variants. It is also observed that the NN model is not suitable for covering attacks that produce lower volume traffic. Most of the approaches mentioned above also have constraints like network source and destination or topology specific. They are not generic models for ZAs prevention. As a whole, it is tough to design a generic model with higher accuracy and lower FAR to defend zero-day HVA and LVA exploits.

These limitations motivate the authors to propose a model that detects high and low volume ZAs with higher performance by applying HH and graph-based approaches. This generic model is independent of any specific assumption regarding source and destination, topology, etc.

**Table 1:** Summary of key-state-of-the-arts

Author & Year	Methodology	Summary
Blaise et al. [44],2020	Statistical approach	$\bullet$Port uses profile based detection
	Based on analysis	$\bullet$Distributed collection of host traffic
	of ports	$\bullet$ Focused on high volume attacks
		$\bullet$Does not cover low volume attacks
R.M. et al. [38],2020	DNN based approach	$\bullet$PCA & GWO for dimensionality reduction and optimization
		$\bullet$ Accuracy is enhanced more than 15% applying dimensionality reduction technique
Javed et al. [40],2020	LSTM based CNN Model	$\bullet$Applied voting scheme to detect abnormality in data generated
		through automotive vehicles on different classifier to make final decision
Sameera & Shashi et al. [41],2020	DNN	$\bullet$Used manifold alignment to get rid of different feature space
		$\bullet$Applied soft labeling to get the label to the unlabeled data
		$\bullet$Zero-day LVA (probe and R2L) detection by using HVAs (DoS) training phase
		$\bullet$NSL-KDD to CIDD ZA detection analysis shows lower performance
Hindy et al. [43],2020	DNN	$\bullet$Proposed optimized DNN architecture for autoencoder to detect ZAs
		$\bullet$Analyzed performance on CICIDS2017 and NSL-KDD data set
Alauthman et al. [45],	Reinforcement learning	$\bullet$Model features are selected using CART
2020	-based detection	$\bullet$ Bots detection
		$\bullet$Evaluated on real-time captured network traffic
Singh et al. [25], 2019	Hybrid approach using	$\bullet$Ranking algorithm assigns the likelihood of exploits based on frequency
	Snort IDS	$\bullet$Builds attack graph for specific time stamps
		$\bullet$Focused on HVAs
		$\bullet$LVAs are not taken into consideration
Tang et al. [39],2019	Statistical Model	$\bullet$Aims to disclose the relation between different vulnerability and exploits
		$\bullet$Used Gaussian and Student-t distribution as copula function
Khan et al. [21], 2019	Hybrid approach using	$\bullet$Captures benign traffic signatures using bloom filter and KNN
	Bloom filter & KNN	$\bullet$Bloom filter poses high FPs
		$\bullet$Detects LVA and HVA ZAs through Anomaly-based approach
Kumar et al. [46],2019	Deep Learning	$\bullet$Malware detection is implemented through static, dynamic and image analysis
	approach	$\bullet$Works on malware executable binaries
		$\bullet$Host-based technique
		$\bullet$Works at kernel level to detect ZAs
Sun et al. [11], 2018	Bayesian networks	$\bullet$Graph nodes consist of instances of file, process etc.
	based approach	$\bullet$Performance depends on availability of accurate evidences
		$\bullet$ Host oriented technique
Kim et al. [37], 2018	GAN based on deep	$\bullet$ Zero-day detection works by adding noise to existing malware
	autoencoder	$\bullet$Fixed length zero-day malware detection
Duessel et al. [17], 2017	One class SVM	$\bullet$Combines protocol context with sequential features
		$\bullet$Covers application layer attacks only

## Risk observations (attack analysis)

Detecting known attacks is comparatively more straightforward due to the availability of signatures in the public domain. On the other hand, exploits whose signatures are unknown to the developer-defined as zero-day exploit or attack. It is tough to design a model with higher accuracy and a lower false alarm rate to defend against these attacks. Security persons or the public are unaware of the attacks that executes on their system. As a result, the cost of penalty can range from moderate to high. The proposed work broadly divides the ZAs into two categories (*a*) Zero-day HVAs and (*b*) Zero-day LVAs. The following subsection describes these two types of ZAs.

### Case 1: zero-day high volume attacks (HVA)

Heavy traffics generated using botnets or by distributed systems encounter HVAs. These attacks include the variants of Dos/DDoS attacks [36, 47–49], where adversaries either try to overflow the objective services or exploit a vulnerability in the software of the server to exhaust system resources and make it inaccessible for the legitimate users. Techniques of performing these types of attacks can be either traffic-based, bandwidth-based or application-based. Zero-day HVAs are those attacks that fall under this category, but the signature or behavior is not available in advance. Thus, it is difficult to capture those attacks because what you don’t know, you can’t predict. DoS/DDoS attack is treated as zero-day if the attack is performed using methods that are not utilized earlier before [50].

*Solution:* Proposed work includes a module to detect zero-day variant of HVAs where two different pools of HVA and non-attack (or normal) traffic are maintained. First, the HVA pool finds the frequent strings present in the pool with an estimated counter value. Again, the same strings are used to find the estimated count in the normal pool. Suppose the estimated counter of a string against the attack and normal pool’s differences exceeds some threshold (discussed in Sect. 5.1.1). The string is stored as an HVA signature in a knowledge base (KB) (for detail, refer to Algorithm 2). Detector passively monitors the real-time traffic for any matching signature generated at the training phase. If any match is detected, the traffic is blocked.

Some genuine events also behave like a variant of DoS/DDoS ZAs. These events are known as flash events [51]. Due to these events, the traffic load on a server suddenly increases. The sudden spike in traffic may result in server failure in the system and inaccessibility to users. These events behave similarly to the HVAs. But, here, the intention of the users to play the discriminating factor. E.g., the traffic load on a university server at the time of results is high. The ticket booking load on railway server in some festive season are also the example of flash events. To avoid such an ambiguous case, plenty of work exists specifically to detect whether the network traffic is due to a flash event or not [51]. If that is the case, the detector module will allow the traffic to pass through. In this paper, it is assumed that a flash event is already a detector which is implemented separately using existing methods [51–55]. It is placed in the network to detect whether traffic load is due to a flash event or HVA.

### Case 2: zero-day low volume attack (LVA)

Apart from HVAs, several other ZAs don’t produce heavy traffic at the victim node, but the consequences can range from mild to severe. Those attacks execute silently to gain access control, data theft, or to perform malicious activities. Generally, variants of backdoor, scanning, generic, etc., are termed as LVAs. These attacks pose unknown patterns or behavior used for information gathering, data theft, etc. There is an exception with the low-volume DoS attack case where it seems to belong to the LVA module, but the HVA module covers it. The main reason behind this is that the proposed framework inherently works on the stream of byte sequence of payloads captured from attack traffic. Few streams in the LVA DoS attack are by default generated through the HVA module. At the time of detection for ZAs, the model works on individual packets independently. Thus, even a DoS attack is getting executed in LVA mode, the HVA module will detect it.

*Solution:* To defend these attack categories, this apaper designs an LVA module that maintains pools of LVAs and non-attack. Now, following the steps similar to the HVA module, LVA strings are generated. The unqualified strings (or tokens) from the HVA module are forwarded to this module. If the forwarded strings are already in the LVA, this module assigns extra weights to the strings already present in the LVA to refer to the higher likelihood of being malicious. Now, a graph is constructed by applying all the strings generated by the LVA module, and each vertex of the graph is assigned a score (Eq. 5) discussed in Sect. 5.1.2. A sequence of vertices (signatures) with a cumulative score higher than the threshold is considered zero-day LVAs signatures. A knowledge base of LVA signatures is produced to accumulate those LVA signatures. Algorithm 3 explains the procedure of signature generation for the LVA module. If any signature from the knowledge base matches the traffic, the detection module triggers an alarm for the malicious traffic.

## Proposed framework

In this section, the proposed framework for detecting ZA consists of two phases-(*a*) signature generation and (*b*) evaluation. The following subsections depict the proposed framework.

### Signature generation phase

This phase consists of two modules where module 1 shows the signature generation for high volume attacks (HVA), i.e., variants of DoS/DDoS attack [36, 47–49] and module 2 shows the signature generation for low volume attacks (LVA) [56]. LVAs include variants of service scanning, data theft, OS fingerprinting etc. Strings obtained at the end of each module are considered as ZAs signatures.

**Fig. 3:**
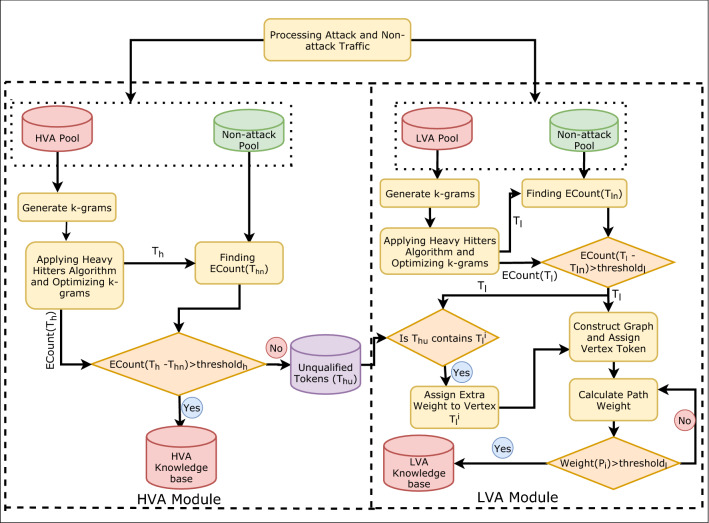
Building signature-base for ZA detection

**Fig. 4:**
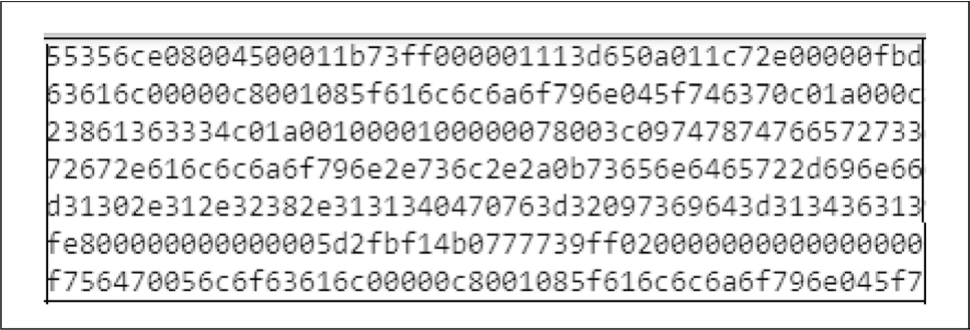
Pool example

Figure 3 shows that each module uses two pools that maintains records in hexadecimal byte format. In the first module, pools consist of HVAs and non-attack records. Non-attack refers to genuine traffic reflecting the normal working environment. The second module, i.e., the LVA module, consists of the LVA pool, including scan, data theft attack and genuine traffic records. Figure 4 shows the example of a pool that stores raw data in a hexadecimal format. Each module is explained in Sects. 5.1.1 and 5.1.2. Section 6 explains the process of generating raw data for each category. Table 2 depicts all the abbreviations used to describe the proposed framework.

**Table 2:** Abbreviations used in algorithms of the proposed work

Notation	Description
*nonatkpool*	Non-attack or Genuine traffic pool
*HVApool*	HVA pool
*hkb*	High volume attack signature knowledge base
$T_{hu}$	Unqualified heavy-hitter tokens
$m\_token[]$	Merged tokens list
$c_{a}$	Count of token in attack pool
$c_{n}$	Count of token in genuine pool
*thresholdH*	Threshold value for HVA signature qualification
*LVAPool*	LVA pool
*adj*	Adjacency matrix of generated graph
$T_{l}$	Merged tokens of LVA module
*score*[]	Array of score value assigned to each vertex in the graph
$\alpha$	Constant value for tuning score value
$W_{A..B}$	Weight of path from vertex A to B
$W_{avg}$	Average path weight
*thresholL*	Threshold value for LVA signature qualification

#### HVA module

**Fig. 5:**
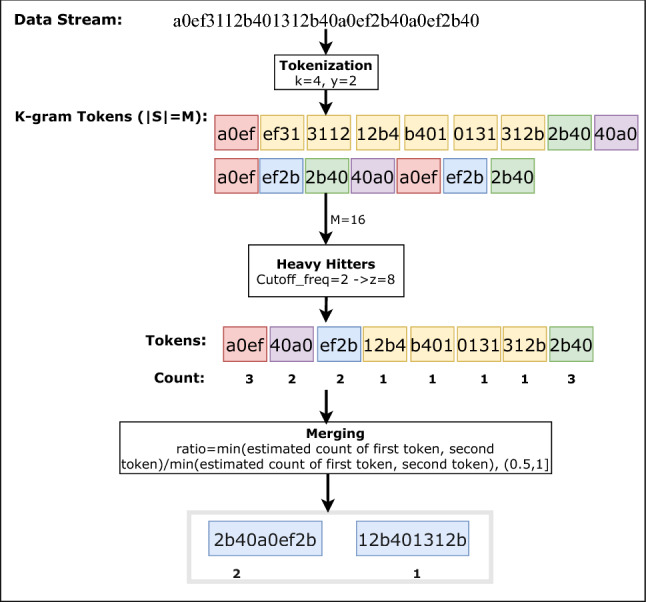
Extraction of tokens from input stream

All signatures present in known attack variations are generated to detect ZA consisting of unknown variants of DoS/DDoS or similar attacks. This module performs all the steps required to create HVAs signatures. It consists of two pools of raw packet byte streams merged in a single text file. Traffic captured through Wireshark in *pcap* format of different attack categories is used to generate variable length frequent string tokens consisting of hexadecimal characters. These strings are subject to merging and optimization that finally pave the way to form the HVA signatures. This process involves a direct extraction of data packet array (i.e., byte stream $\sigma$) from *pcap* file. After removing the redundant information, all packets are merged into one file. This $\sigma$ is used to extract the tokens of size *k* by sequentially sliding the *k-gram* window with a fixed step size *y*. Fig. 5 shows this process. Hence, tokens initially represent the k-grams of a byte stream and store it in a file *S*.

Suppose *m*, *k* and *y* are the size of a raw stream, token length (or window size) and sliding step size, respectively. These variables are always of even length because each byte of data packets is converted to its hexadecimal representation. Here, *y* is always less than *k*. So,in byte form, the stream size becomes *m*/2 byte, window size becomes *k*/2 and similarly, the step size becomes *y*/2. As a whole, the total number of tokens possible in byte form is $\{m-(k-y)\}/2$.

E.g., let’s assume the stream shown in Fig. 5. We need to extract all 4-grams of this stream. Now referring to the figure, the first 4-gram is *a0ef* and is represented by the first red box. The others are described in sequence within individual boxes by sliding the window one byte, i.e., two hexadecimal characters to the right.

Following procedure describes extraction of HVA token:

- After extracting all k-grams (i.e., 16), the heavy-hitter algorithm with cutoff frequency (i.e., 2) is applied to derive all the frequent tokens (or k-grams) (i.e., 8) present in S.
- z is calculated using Eq. 2.
  2 $$\begin{aligned} z=\lceil \frac{M}{\mathrm{cutoff}\_\mathrm{freq}}\rceil \end{aligned}$$
  where M is the size of tokens set, frequency indicates a constant value for the minimum occurrence of each token, and z is the ceil value of the ratio of the number of tokens M and the frequency($cutoff\_freq$). The values of M and frequency are considered as 16 and 2, respectively, for the above example.
- Algorithm 2 guarantees that the z frequent tokens obtained from S must contain the top frequent k-grams. But the converse is not valid, i.e., z can contain k-grams which have a frequency less than the cutoff frequency.
- Finally, those top *k* tokens are merged to reduce the redundancy, which generates variable-length tokens.
- Merging of tokens refers to the process, where two frequent tokens found by the Algorithm 2 which have some parts matching from either end of the token. They fall within some specified ratio(r) (given by Eq. 3) of estimated occurrence with the other.
  3 $$\begin{aligned} r=\frac{\mathrm{min(estimated}\ \mathrm{count}\ of\ \mathrm{first}\ \mathrm{and}\ \mathrm{second}\ \mathrm{token)}}{\mathrm{max(estimated}\ \mathrm{count}\ \mathrm{of}\ \mathrm{first}\ \mathrm{and}\ \mathrm{second}\ \mathrm{token)}} \end{aligned}$$
  From Fig. 14, the value of r is assumed $\in$(0.5,1].
- The newly merged token is placed on the list and the count is set to the minimum of the estimated counter of individual tokens.
- It checks whether any existing token contains in the merged token or not. If it is found, the existing token is removed from the list.
- Now, rather than directly using these merged token lists as attack signatures, each one is validated using HVA and non-attack pool.
- If the count of token in HVA pool is higher than that of in non-attack pool by thresholdH given by Eq. 4, that token is kept in HVA knowledge base as the final signature. Otherwise, the token is considered as a non-HVA token set ($T_{hu}$).
- The threshold thresholdH is computed using Eq. 4.
  4 $$\begin{aligned} \mathrm{thresholdH}=\theta *N \end{aligned}$$
  Where $\theta$ $\in$[0,1] and N is the total number of tokens.
- The best value of $\theta$ is decided by plotting the ROC curve for various attacks for different value of $\theta$ (shown in Fig. 14 of Sect. 7).
- After completing the execution of this module, non-HVA token ($T_{hu}$) is passed to LVA module.

Algorithm 2 describes the concept of deriving HVA signatures where $extract\_Token(\sigma ,k)$ is a method used to extract all possible tokens from input stream $f\_tokenh[1\ldots z]$. It is the list of top z frequent tokens. The $merge\_Token(\sigma ,f\_token[])$ method is used to merge tokens obtained by $extract\_Token()$ method and returns the merged frequent token ($f\_token$) array.
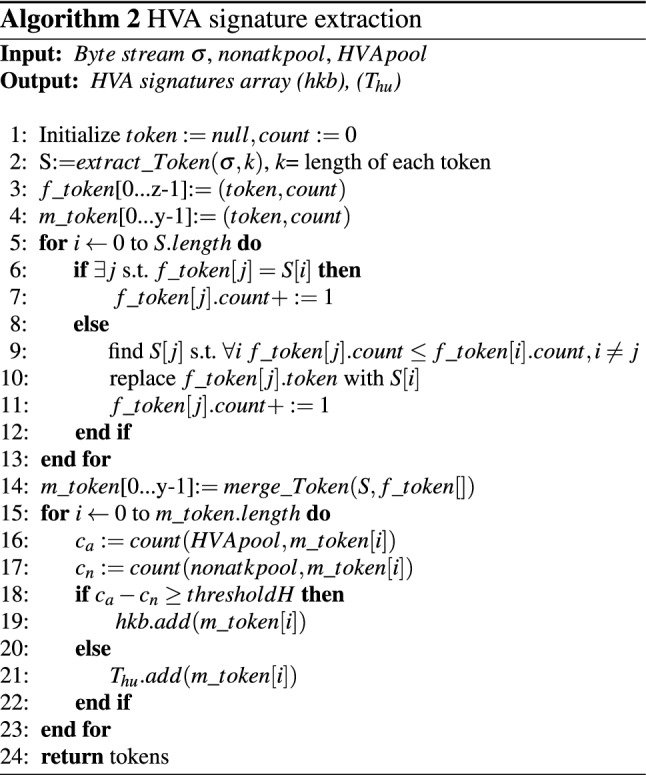


#### LVA module

**Fig. 6:**
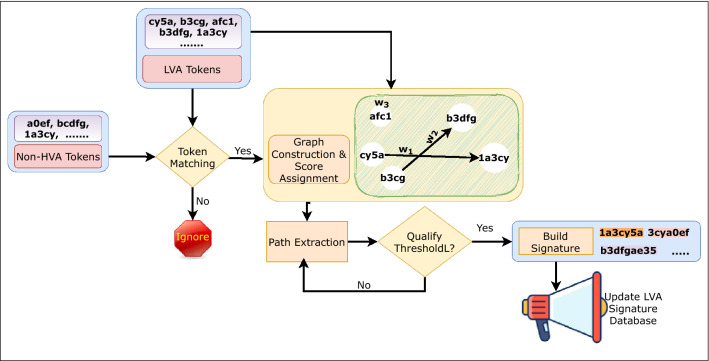
Advancing of LVA signature extraction phase

This module generates signatures for low-volume ZAs. It takes non-HVA tokens ($T_{hu}$) from the HVA module and generates k-grams. Those k-grams are merged by following the similar process as in the HVA module to produce a set of tokens $T_{l}$. The zero-day LVA attack signature extraction procedure is shown in Fig. 6.

The signatures are generated in the following way:

- Frequent tokens are found in the LVA module by applying the same procedure as in the HVA token generation and is used to construct a graph.
- These frequent tokens are used as vertices of the graph and the edges between them represent the consecutive occurrence of those tokens in attack pool.
- Score is assigned to each vertex by a score function *vscore*() given by Eq. 5. It takes *x*, *aPool*,  and *nPool* as input.
  5 $$\begin{aligned} \mathrm{vscore}(x, \mathrm{aPool}, \mathrm{nPool})=\frac{\mathrm{fraction}\ \mathrm{of}\ x\ \mathrm{in}\ \mathrm{aPool}}{\mathrm{fraction}\ \mathrm{of}\ x\ \mathrm{in}\ \mathrm{nPool}+1} \end{aligned}$$
  where, *x* is a token from array *a*[] representing a vertex. The *aPool*, and *nPool* represent attack pool and normal pool respectively. The numerator and denominator are the proportion of token *x* in attack and normal pool calculated by Eqs. 6 and 7.
  6 $$\begin{aligned} \mathrm{fraction}\ \mathrm{of}\ x\ \mathrm{in}\ \mathrm{aPool}=\frac{\mathrm{count}(x)\ \mathrm{in}\ \mathrm{aPool}}{\varSigma _{\forall \ a[i]} \mathrm{count}(\mathrm{a[i]},\mathrm{aPool})} \end{aligned}$$
  7 $$\begin{aligned} \mathrm{fraction}\ \mathrm{of}\ x\ \mathrm{in}\ \mathrm{nPool}=\frac{\mathrm{count}(x)\ \mathrm{in} \ \mathrm{nPool}}{\varSigma _{\forall \ a[i]} \mathrm{count}(a[i],\mathrm{nPool})} \end{aligned}$$
  Count refers to the estimated count produced by heavy hitter algorithm.
- Every qualified tokens are checked against the $T_{hu}$. Any token contained in $T_{hu}$ is assigned some extra weight (refer to Algorithm 3) to reflect higher likelihood of being malicious for that vertex.
- The coefficient $\alpha$ in Algorithm 3 denotes a value in range [0,1].
- The weight of the path represents the average path score from one vertex to another and is calculated using Eq. 8.
  8 $$\begin{aligned} w_{A...B}=\frac{S_{A}+...+S_{B}}{\mathrm{number}\ \mathrm{of}\ \mathrm{vertices}\ \mathrm{involved}\ \mathrm{in}\ \mathrm{path}\ AB} \end{aligned}$$
  Where, $w_{A...B}=weight\ of\ path\ AB$, and $S_{A},\ S_{B}$ are the scores of vertices involved in path A to B.
- Based on the weight of the path or by using the score itself in case of isolated vertices, the final signatures are extracted by setting up appropriate threshold thresholdL given by Eq. 9. The vertices of a path that qualifies the threshold are merged to represent LVA signatures. They are used to detect zero-day LVA attacks.
  9 $$\begin{aligned} \mathrm{thresholdL}=\theta *W_{\mathrm{avg}} \end{aligned}$$
  Where, $W_{avg}$ is the average weight of all the edges and calculated by Eq. 10.
  10 $$\begin{aligned} W_{\mathrm{avg}}=\frac{\mathrm{sum}\ \mathrm{of}\ \mathrm{all}\ \mathrm{weights}\ \mathrm{of}\ \mathrm{a}\ \mathrm{path}}{\mathrm{total}\ \mathrm{number}\ \mathrm{of}\ \mathrm{edges} } \end{aligned}$$

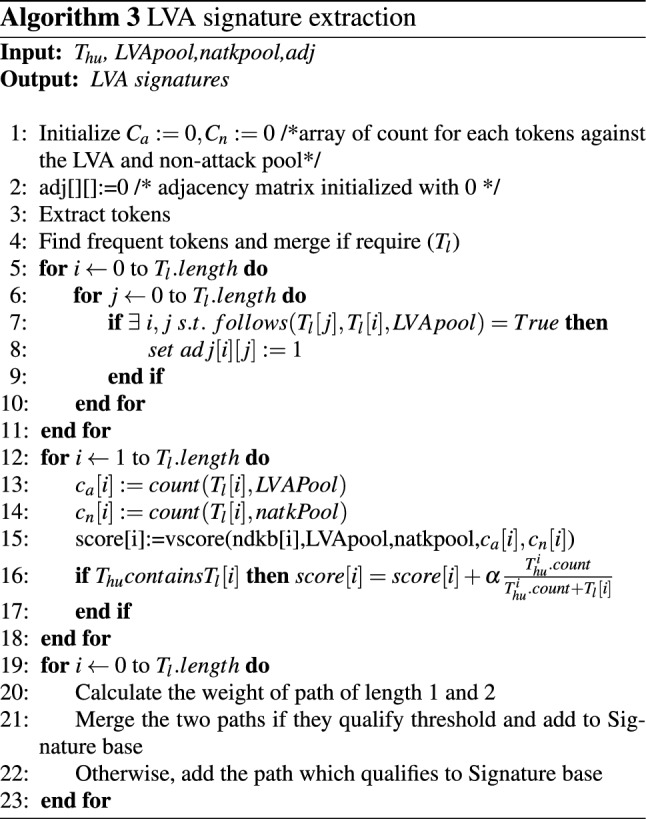

**Fig. 7:**
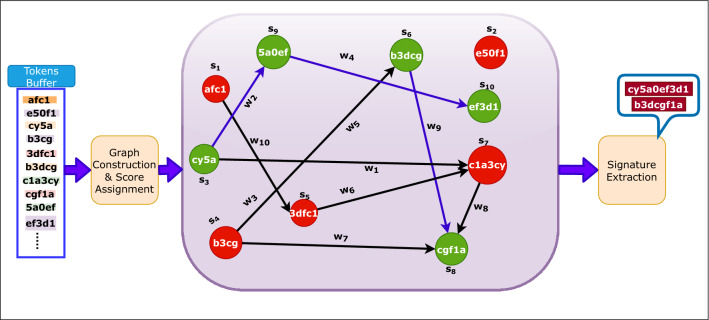
Analogy graph used in the proposed work

Let’s assume that in Fig. 7 the vertices with green color indicate the vertices that qualify for signature. The edges between them are indicated by blue color and are assigned with some weight $w_{i}$. These directed edges between vertices show an ordering relation among them along with the possible matching eat either end of tokens. For e.g., $s_{3}\rightarrow s_{9}\rightarrow s_{10}$ and $s_{6}\rightarrow s_{8}$ showing the qualified paths for attack signatures. The vertices are merged for the selected paths, referring to the zero-day LVA attack’s final signature. As a result, the final signature of zero-day LVA attack at any time instant includes “cy5a0ef3d1” and “b3dcgf1”.

### Attack detection Phase

After completion of the signature generation phase, the next stage is the attack detection phase. The system generates a knowledge base consisting of attack signatures. These signatures represent the lowest unit which could be a significant deciding factor of whether a flow is malicious. This phase consists of attacks signature knowledge bases, a data capturing and preprocessing unit and an attack detection unit. The detection module takes real-time traffic as input and transforms the data into requisite form to detect ZA traces using knowledge bases. Even if one signature matches the flow, the system generates the alarm to the security experts for further analysis and temporarily blocks that flow. If the expert confirms the attack, the model uses that traffic to discover other new signatures.

**Fig. 8:**
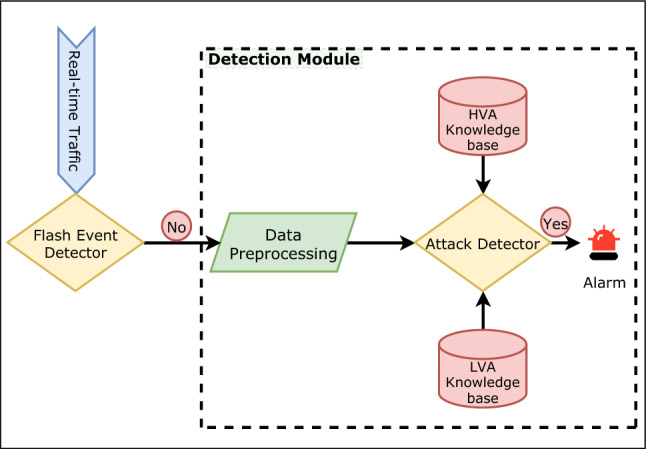
Detecting ZA by applying signature knowledge base

Now to avoid the ambiguity between the signatures present in both flash events and actual attack events, a complementary module for detecting flash events (discussed in Sect. 4.1) is implemented before the data processing unit. This flash event module helps to reduce the FAR. This module first checks any incoming traffic is a flash event or not. If it finds any flash event on the network, network load sharing or other techniques [51–55] is triggered to prevent the network failure. Otherwise, the traffic is sent to the detection module for any ZA detection. If the flash event detector is not there, traffics like connection requests to a particular service may show a similar surge in the case of HVAs. E.g., simultaneous TCP syn requests from a vast number of legitimate users on a network can mimic the SYN flood attack. Thus, the detection module may detect it as attack traffic wrongly. As a result, it increases False Alarm Rate (FAR). Flash Event detector removes this problem. Figure 8 describes the attack detection phase with flash event detection.

In the proposed work, we aim to detect the ZA and achieve this; the test data set comprises a different set of attacks that are not present in the signature generation phase. The basic idea behind it is that every new attack inherently poses a few known attack patterns that act as the critical factor for detecting a ZA [11]. Algorithm 4 discusses the process to generate the detection matrix used to analyze the system’s performance. Input to the algorithm is the array of signatures (i.e., *sign*[]), packets (i.e., *p*[]) and the output is an array of detected packets *d*[], where the indices represent the packet number. The corresponding value is the count of signatures found for each packet. The value zero for any index indicates that the packet is not malicious, i.e., it belongs to a normal category. A value greater than zero indicates the packet is malicious.
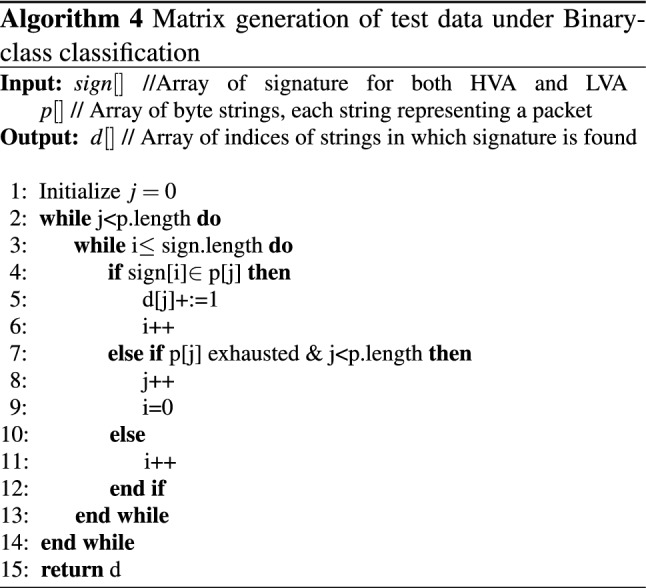


For multiclass classification, the Algorithm 4 is slightly modified to generate the detection matrix for multiclass (i.e., HVA, LVA, & Normal) by providing separate input packets for each class and applying signatures separately for HVA and LVA to each input class. The algorithm for multiclass generates two output arrays $d_{1}, d_{2}$ for each attack class HVA and LVA respectively and is explained in Algorithm 5.
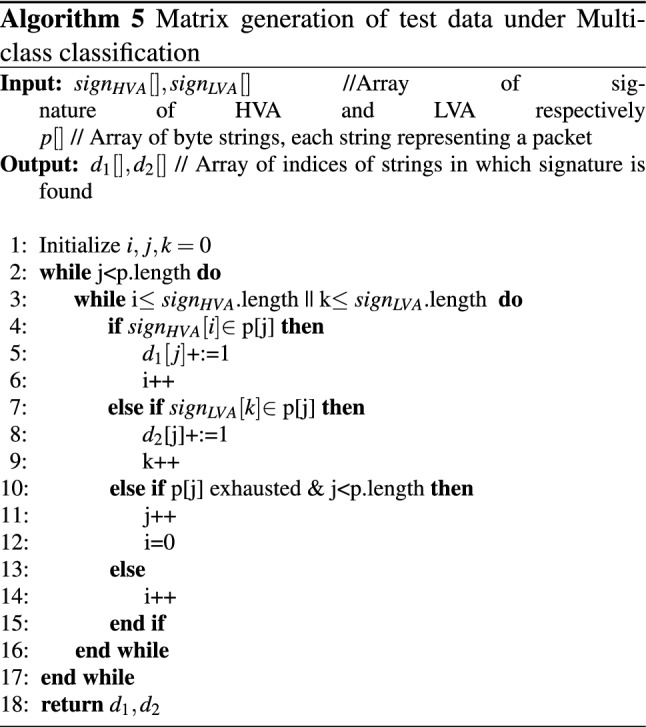


### Working procedure

**Fig. 9:**
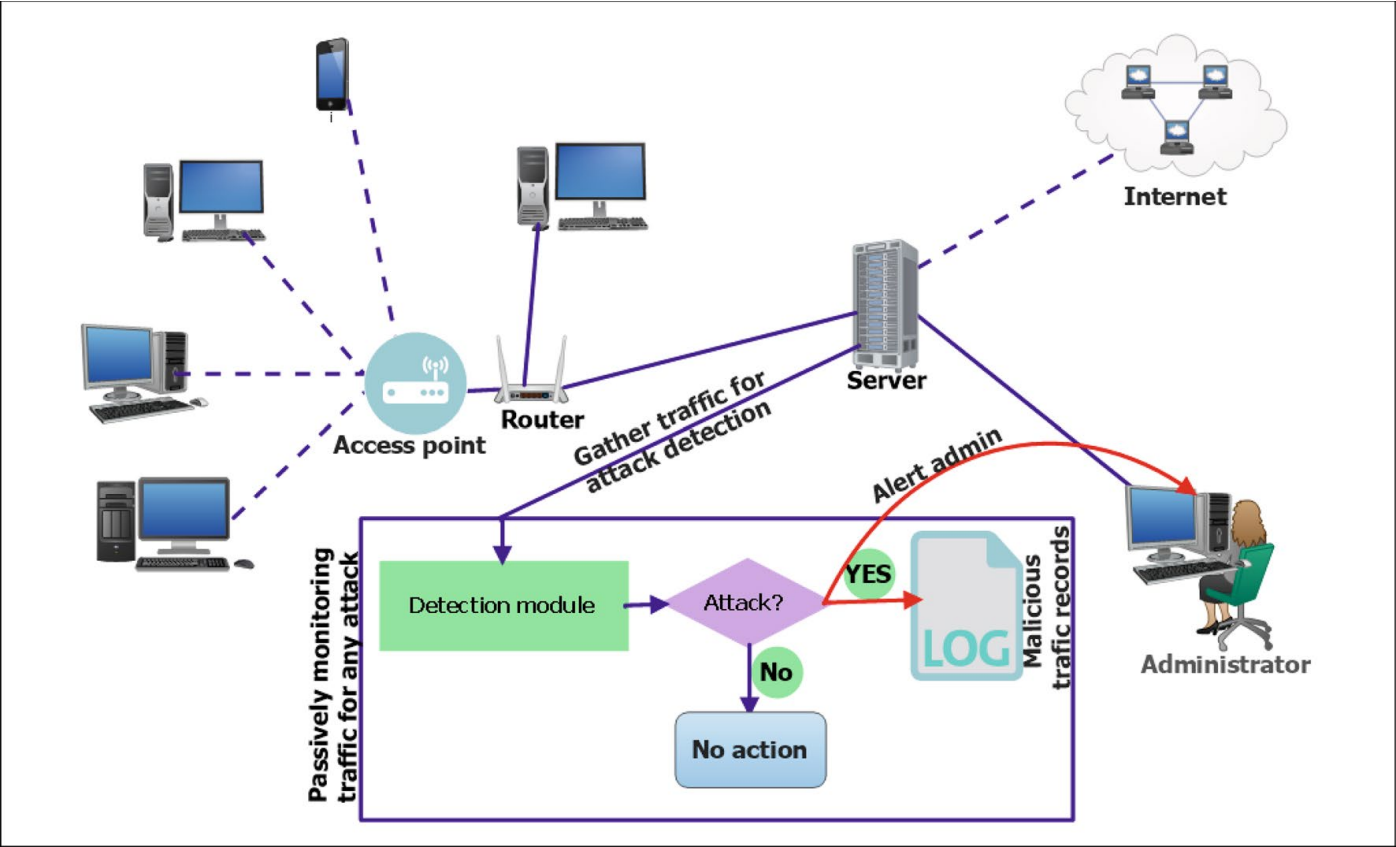
Working procedure of the proposed work

The proposed work’s working procedure is shown in Fig. 9, where the detection module is placed on the server. A copy of all the communication traffic goes through this module. The collected copy of the traffic is preprocessed and fed into the detection module, which is responsible for detecting malicious traffics. The records corresponding to the detection of malicious activities are logged. Also, an alert for the same is sent to the administrator to validate it.

## Experimental setup

For demonstrating the efficiency of the proposed work in detecting ZAs, the experimental setup is divided into two parts.

### Real-time data set

Here, the data set is generated by setting up a virtual environment. The setup consists of 10 genuine, 10 HVA, and 5 LVA nodes to generate traffic. Table 3 lists system specification of individual application and platform used in the proposed work.

**Table 3:** System Specification

Operating System	Version	System Specification
Kali Linux 64-bit	2020-3, 2019-4	**CPU:** Intel Core i5 Processor
Ubuntu Server/Client	14.04.5 LTS, 16.04 LTS	**:** Intel Core i3 Processor
Metasploitable	v4.11.4-2015071402	**RAM:** 4/8 GB DDR4 2400
Windows 32/64-bit	NT 6.1, NT 6.3, NT 10.0	**HDD:** 500 GB/ 1 TB SATA

**Fig. 10:**
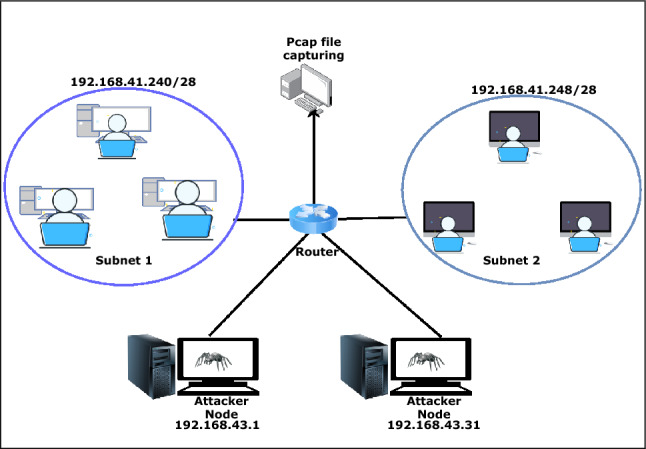
System setup to generate data for the proposed work

**Fig. 11:**
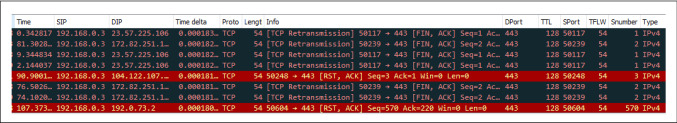
Wireshark window capturing DoS attack

The data generated through this virtual environment consists of the variation of the attack categories considered in the signature generation phase. A setup consisting of Kali Linux, ubuntu server machine and client ubuntu shown in Fig. 10. Here, ubuntu server/client, windows and metasploitable act as victim nodes. The Linux operating system acts as an attacker node. It provides several inbuilt tools to perform different attacks. But in some cases, we have to use python scripts to perform attacks. All the generated traffic is captured through the Wireshark tool. An example of data capturing through Wireshark shown in Fig. 11. The attack performance procedures for DoS/DDoS, probe, data exfiltration and keylogging attacks are discussed in the following subsections.

**Fig. 12:**
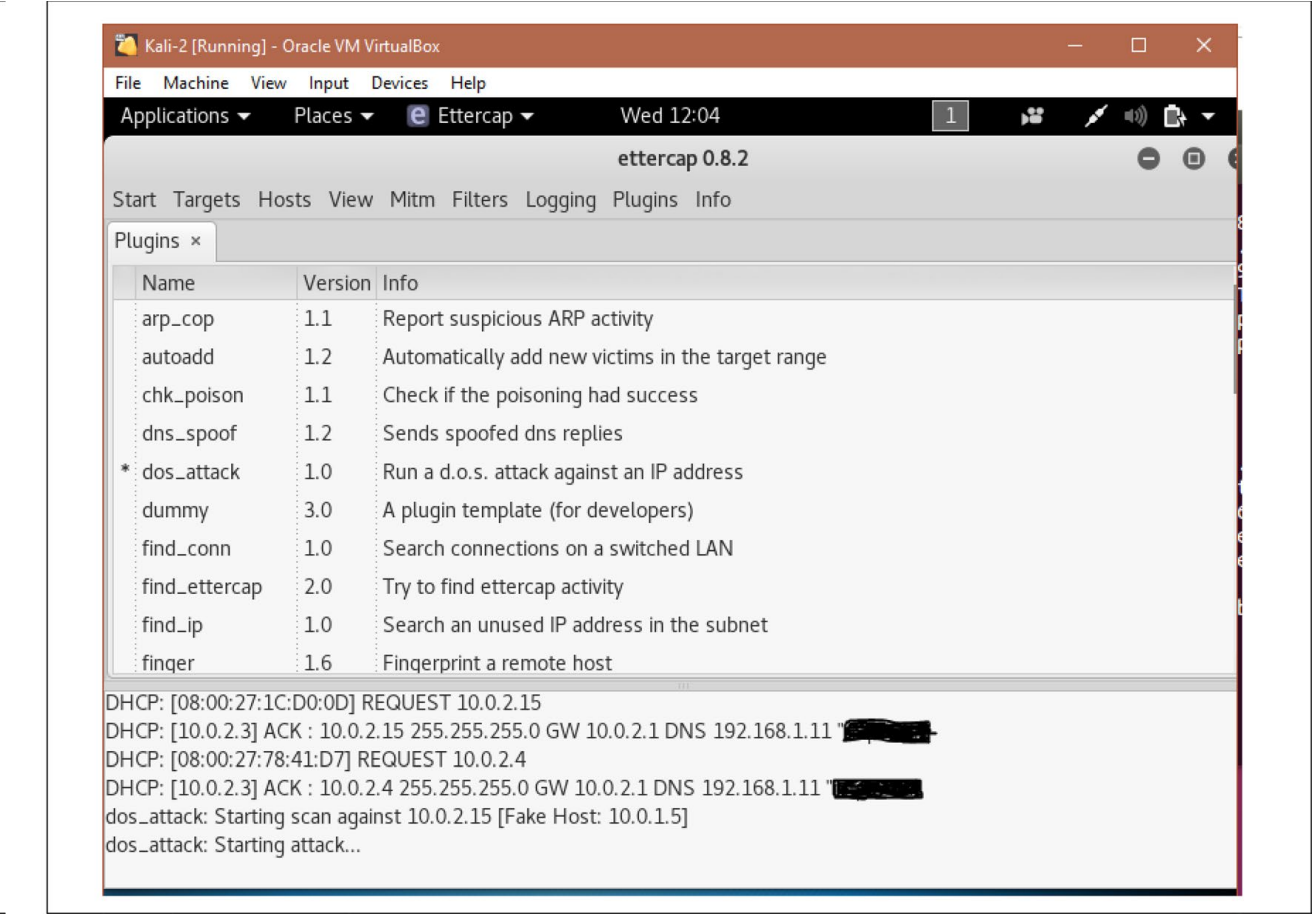
Performing DoS attack using ettercap

**Fig. 13:**
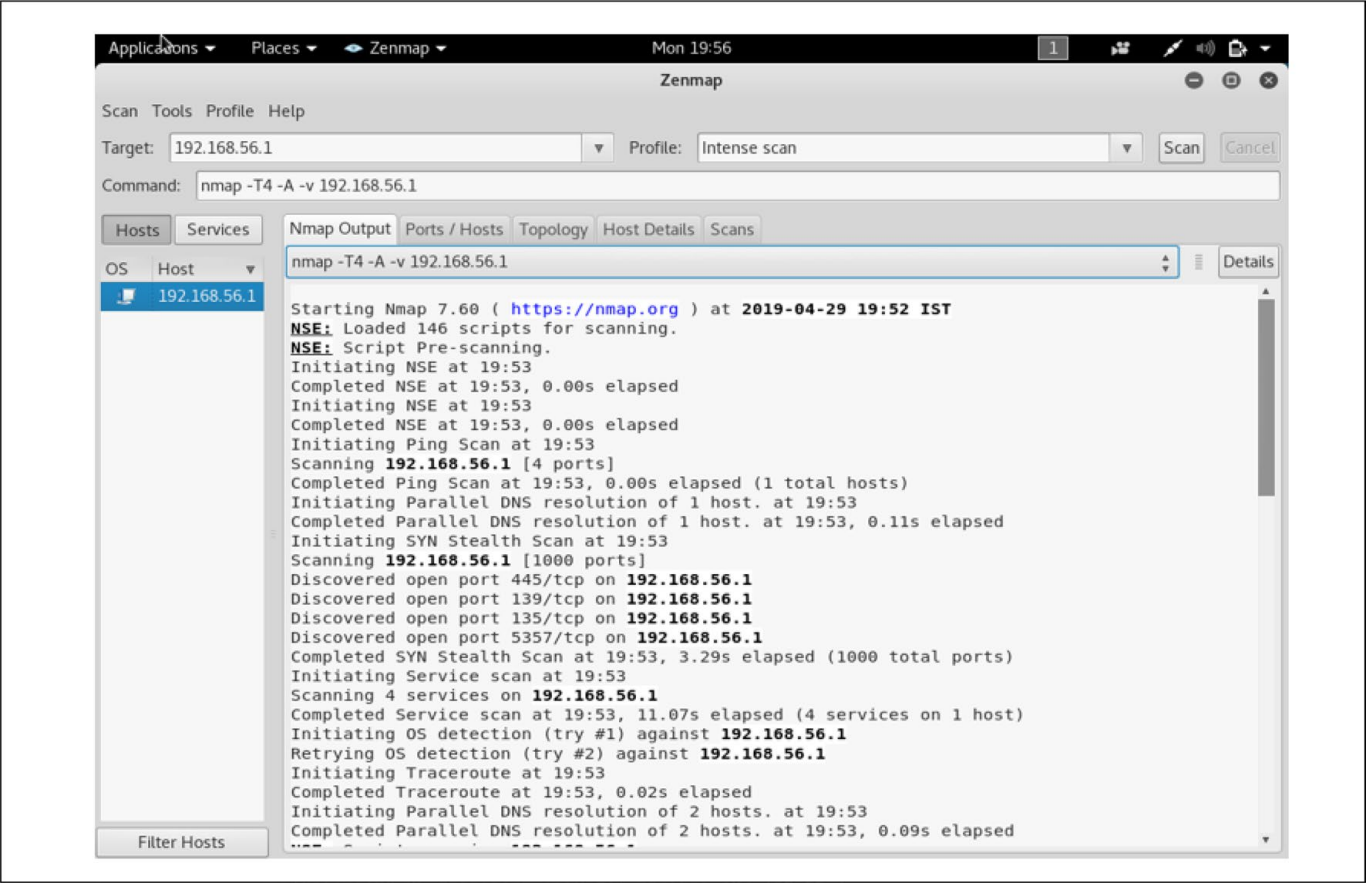
Performing probe on a target host

*A.*: *DoS/DDoS attack*
  This attack is performed for three different categories: HTTP, TCP, and UDP, through other techniques available in the Kali Linux platform, e.g., Ettercap, Metasploit framework SlowHTTPTest, etc. Figure 12 shows the snapshot of running a DoS attack. The procedure to perform the attack through Ettercap is explained below.
  −: Open terminal and type - $sudo\ ettercap - G$
  −: Select sniff menu under that select unified sniffing
  −: In the pop-up window, go to the plugin
  −: Choose the appropriate option to start the attack.
*B.*: *Probe* This attack is used to find all the open ports, system information. Different operating systems running, etc. Zenmap, a popular GUI-based scanning tool in Kali Linux, is used to perform this attack through which OS, service, and other information from destination hosts are collected. Figure 13 shows a snapshot of performing probe to a particular host “192.168.56.1”.
*C.*: *Data exfiltration* To perform the attack, cloakify-factory [57] a python script is used, works in several steps which are as follows:
−: Run the python script
−: Select file option
−: Specify the source file path
−: Specify the destination file path to save the output
−: Select the ciphering option to convert the file into ciphertext and to add noise
*D.*: *Keylogging* To perform the keylogging attack, Beelogger [58], a python script is used. All the data is captured by the Wireshark at the victim’s side and stored in a .pcap file. These files are then processed to evaluate the proposed work explained in Sect. 5.2.

There is no way to test the ZA on the run because those attacks are precise to particular zero-day vulnerabilities and not known or just disclosed to a fraction of users. Demonstration of the working of proposed method against ZAs assumes that, at a specific instance of time, known attacks are available for the signature generation phase. On the other hand, a set of attacks absent in the signature generation phase are assumed to be ZAs. It is believed that they are not known to the system at that particular time instance. The distribution of attack data in training and testing is shown in Table 4.

### CICIDS18 benchmark data set

We have additionally used a subset of the latest benchmark data set CICIDS18 in pcap format for two different days covering bruteforce and DDoS attacks. The purpose of selecting these two attack traffic is that both these attack categories are not considered at the signature generation phase. The number of normal traffic packets (6032) is kept the same as earlier during the real-time analysis. The DDoS pcap file used from this data set is different from how DDoS attack is generated through virtual setup during real-time traffic capturing in the proposed work. The number of packets in the HVA and LVAs is 273605 and 69656, respectively, for analysis. Now, the performance of the model is evaluated on both real-time and the CICIDS18 benchmark data set.

**Table 4:** Class distribution among training and testing data

Attack Type	Training Data	Testing Data
TCP SYN	Yes	No
UDP Flood	No	Yes
HTTP Flood	Yes	No
Probe	Yes (Service scan)	Yes (OS,Network scan)
Data Theft	HTTP	FTP

## Performance analysis

In this section, the performance of the proposed approach is discussed for the binary and multi-class test scenarios. At the first time and space complexity of the proposed model is discussed in the following subsection.

### Complexity analysis

The summary of abbreviations used to analyze the time and space complexity of the proposed work is listed in Table 5. This analysis is divided into four phases.

**Table 5:** Abbreviations used for the complexity analysis

Notation	Description
$T_{H}$	Number of tokens generated using HVA pool
$T_{L}$	Number of tokens generated using LVA pool
$T_{N}$	Number of tokens generated using genuine pool
$P_{H}$	Number of packets in HVA pool
$P_{L}$	Number of packets in LVA pool
$P_{N}$	Number of packets in genuine traffic pool
*N*	Total number of tokens extracted from traffic
*z*	Number of frequent tokens
$T_{s}$	Total signatures
*E*	Edge set
*l*	LVA signatures
*T*	Vertex set comprising of LVA qualified tokens
*p*	number of test packets

- *Data Collection:*
  a *Time Complexity*: In the proposed work total data packet is captured on the run-time through virtual setup. Hence, the time complexity for this is nearer to the real-time required for capturing the data packets.
  b *Space Complexity*: The storage complexity is proportional to store the HVA, LVA and genuine packets in pools. It is in the order of $O(P_{H}+P_{L}+P_{N})$, where $P_{H}, P_{L}$ and $P_{N}$ are defined in 5.
- *HVA Signature Generation:* Algorithm 2 explains HVA signature generation.
  - *Time Complexity*
    i. Token Extraction: According to algorithm 2, extracting possible number of tokens from merged input stream of all packets with *m* characters, *k* token length and *y* sliding step size is $m-(k-y)$. Hence, the time taken for execution is the linear order of *m* i.e., *O*(*m*).
    ii. Finding top z frequent tokens from m-(k-y) is bounded by the size O(m). Hence, replacing the element with minimum count to find the top z token applying Min heap sorting requires $O(z\log (z) )$ time. Hence, the total time require is $O(m*z\log (z) )$.
    iii. Token optimization: Merging of tokens that matches patterns at either end by applying LCP merge takes $O(z \log (z))$.
    iv. Finally, finding heavy hitters will take time *O*(*z*).
    Hence, the overall time complexity for algorithm 2 is:
    $O(m)+O(m*z\log (z))+O(z \log (z))+O(z)$, which is the time complexity of HH signature generation.
  - Space Complexity: For maintaining pool to store traffic requires storage of order $O(P_{H}+P_{N})$ and *O*(*z*) to store the z frequent tokens. So, the overall space complexity of the algorithm is: $O(P_{H}+P_{N}) + O(z)$
- *LVA Signature Generation:* Algorithm 3 describes the process of LVA signature generation process.
  - *Time Complexity*
    i. Extracting Tokens: Similar to the Algorithm 2, extracting and optimization of tokens take $O(m)+O(z\log (z))$ time.
    ii. Creation of adjacency matrix with *z* tokens requires $O(z^{2})$ time.
    iii. The assignment of score of the *z* vertices of the graph require a time of $O(z*(T_{L}+T_{N}))$.
    iv. Creating a signature by assigning weight to each path and checking each path for threshold qualification is a graph traversal that takes $O(E+T)$.
    Hence, the overall time complexity of Algorithm 3 is: $O(m)+O(z\log (z))+O(z^{2})+O(z*(T_{L}+T_{N}))+O(E+T)$
  - *Space Complexity*: Storage require to store frequent token is *O*(*z*), adjacency matrix is $O(z^{2})$ and for LVA signature O(l).
  Hence, the overall space complexity of the algorithm is: $O(z)+O(z^{2})+O(l)$
- *Performance Metrics:* Algorithm 4 and 5 specifies the matrix generation for binary and multi-class classification to evaluate the performance of the proposed model.
  - *Time Complexity*: Generating detection metric takes $O(T_{s} *m)$ time.
  - *Space Complexity*:Since the matrix keeps the count of the number of tokens matching with each packet *p*, space complexity to store the detection matrix is *O*(*p*).

### Determination of $\theta$ for signature extraction

**Fig. 14:**
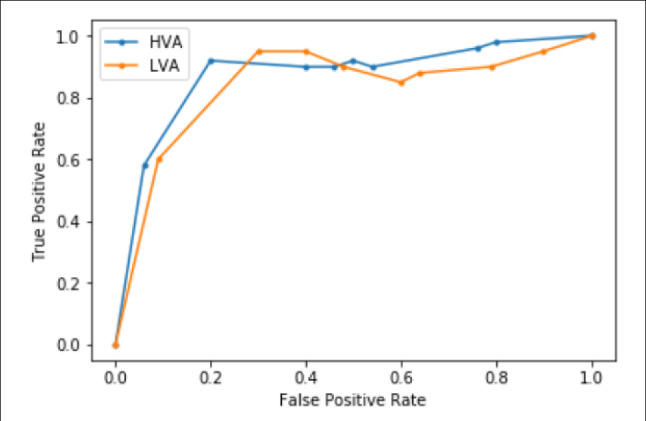
Roc curve to decide the best value of $\theta$

Now, before analyzing the performance of the proposed work, it is necessary to identify the best possible value of $\theta$ (see Eqs. 4 and 9), which is used to extract signatures. Several values of $\theta$ are used to select HVA and LVA signatures. A ROC curve (Fig. 14) shows the (FPR, TPR) pair value for different thresholds obtained by varying the value of $\theta$. FPR and TPR are also known as FAR and Recall. The $\theta$ corresponding to the point, which shows the best result of TPR and FPR in the ROC curve, is selected for the proposed framework. $\theta$ for HVA is considered as 0.5 and for LVA, it is 0.7. In Fig. 14 these values are corresponding to the FPR range [0.2,0.4) and TPR range (0.8,1.0).

### Performance metrics

The performance metrics used to evaluate the proposed work are discussed below for binary classification (*bin*) and multi-class classification (*mul*).

- *Accuracy:* It is the measure of correctness in the detection of different classes to the total input given to the system and is given by Eqs. 11a and 11b.
  11a $$\begin{aligned} \mathrm{Acc}_{\mathrm{bin}}&=\frac{\mathrm{TP}+\mathrm{TN}}{\mathrm{TP}+\mathrm{TN}+\mathrm{FP}+\mathrm{FN}} \end{aligned}$$
  11b $$\begin{aligned} \mathrm{Acc}_{\mathrm{mul}}&=\frac{\mathrm{packets}\ \mathrm{correctly}\ \mathrm{classified}}{\mathrm{Total}\ \mathrm{number}\ \mathrm{of}\ \mathrm{packets} } \end{aligned}$$
- *Recall:* It is defined as the number of packets of each class predicted correctly over the total packets of that class and is mathematically calculated by Eq. 12a and 12b.
  12a $$\begin{aligned} \mathrm{recall}_{\mathrm{bin}}&=\frac{\mathrm{TP}}{\mathrm{TP}+\mathrm{FN}} \end{aligned}$$
  12b $$\begin{aligned} \mathrm{recall}_{\mathrm{mul}}&=\frac{\mathrm{correctly}\ \mathrm{predicted}\ \mathrm{instances}\ \mathrm{of}\ \mathrm{input}\ \mathrm{class} }{\mathrm{Total}\ \mathrm{instances}\ \mathrm{of}\ \mathrm{input}\ \mathrm{class} } \end{aligned}$$
- *Precision:*It is defined as the number of predicted packets that are correct over the total predicted packets for each class and is given by Eq. 13a and 13b.
  13a $$\begin{aligned} \mathrm{Precision}_{\mathrm{bin}}&=\frac{\mathrm{TP}}{\mathrm{TP}+\mathrm{FP}} \end{aligned}$$
  13b $$\begin{aligned} \mathrm{precision}_{\mathrm{mul}}&=\frac{\mathrm{correct}\ \mathrm{instances}\ \mathrm{in}\ \mathrm{predicted}\ \mathrm{class} }{\mathrm{Total}\ \mathrm{instances}\ \mathrm{of}\ \mathrm{predicted}\ \mathrm{class} } \end{aligned}$$
- *F-measure:*It shows the balance between the precision and recall of each class and is given by Eq. 14. We have used the mean F-measure (MFM) for multiclass classification by taking the average of F-measures for all classes.
  14 $$\begin{aligned} \mathrm{F-measure}=\frac{2*\mathrm{precision}*\mathrm{recall}}{\mathrm{precision}+\mathrm{recall}} \end{aligned}$$
- *False alarm rate (FAR):*It is the rate of false alarm generated by the model for non-attack data and is calculated by Eq. 15a and 15b.
  15a $$\begin{aligned} \mathrm{FAR}_{\mathrm{bin}}&=\frac{\mathrm{FP}}{\mathrm{FP}+\mathrm{TN}} \end{aligned}$$
  15b $$\begin{aligned} \mathrm{FAR}_{\mathrm{mul}}&=\frac{\mathrm{misclassified}\ \mathrm{instances}\ \mathrm{of}\ \mathrm{genuine}\ \mathrm{class} }{\mathrm{Total}\ \mathrm{instances}\ \mathrm{of}\ \mathrm{genuine}\ \mathrm{class} } \end{aligned}$$

There are other metrics mainly used for multiclass classification are- average accuracy, attack accuracy, and attack detection rate (ADR) discussed below.

- *Average accuracy (AvgAcc):* It is the average of recalls of all classes and is given by Eq. 16.
  16 $$\begin{aligned} \mathrm{AvgAcc}=\frac{\varSigma _{\forall \ \mathrm{class}\ i }\mathrm{recall}_{i}}{\mathrm{Total}\ \mathrm{number}\ \mathrm{of}\ \mathrm{classes}} \end{aligned}$$
- *Attack accuracy (AttAcc):* It is an average of recalls of all classes except the genuine (normal) class and is given by Eq. 17.
  17 $$\begin{aligned} \mathrm{AttAcc}=\frac{\varSigma _{\forall \ \mathrm{class}\ i \ \mathrm{except for}\ i=\mathrm{genuine} }\mathrm{recall}_{i}}{\mathrm{Total}\ \mathrm{number}\ \mathrm{of}\ \mathrm{classes}-1} \end{aligned}$$
- *Attack detection rate (ADR): *It is the rate of correct prediction of attack categories excluding the normal category and is given by Eq. 18.
  18 $$\begin{aligned} \mathrm{ADR}=\frac{\mathrm{correct}\ \mathrm{predicted}\ \mathrm{instances}\ \mathrm{of}\ \mathrm{attack}\ \mathrm{classes}}{\mathrm{Total}\ \mathrm{instances}\ \mathrm{of}\ \mathrm{attack}\ \mathrm{classes}} \end{aligned}$$

### Performance evaluation

*On Real-time data set:* According to the proposed framework discussed in Sect. 5 and the experimental setup to generate synthetic test data, the system is evaluated using unknown attack categories, which refer to the variant attacks not used in the signature generation phase. The distribution of test data sets under different categories is shown in Table 6. In the proposed work, the HVA class consists of DoS and DDoS variants and LVA has other attack variants except those present in HVA, and finally, the Normal class consists of benign packets.

**Table 6:** Distribution of instances in different classes

Classes	DoS	DDoS	OS scan	Network scan	Data theft	Normal
Instances	5000	4000	5000	3400	1600	6032

**Fig. 15:**
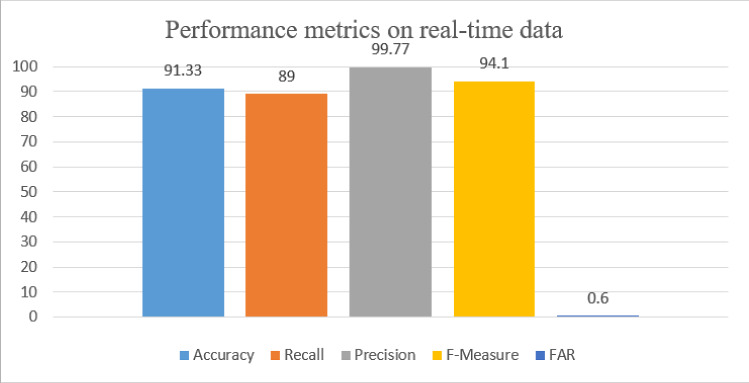
Performance evaluation of the proposed system for binary class

The Confusion matrix for binary classification is shown in the Table 7. The first category is the attack category that includes all attack categories and the other is the normal category. The metrics corresponding to the Table 7 are shown in Fig. 15 with accuracy 91.33% and lower FAR of only 0.6% and optimal values for the precision recall is 99.77% and 89% respectively.

**Table 7:** Confusion matrix for real-time test data set under binary-class classification

Classes	Attack	Normal
Attack	16867	2133
Normal	38	5994

**Fig. 16:**
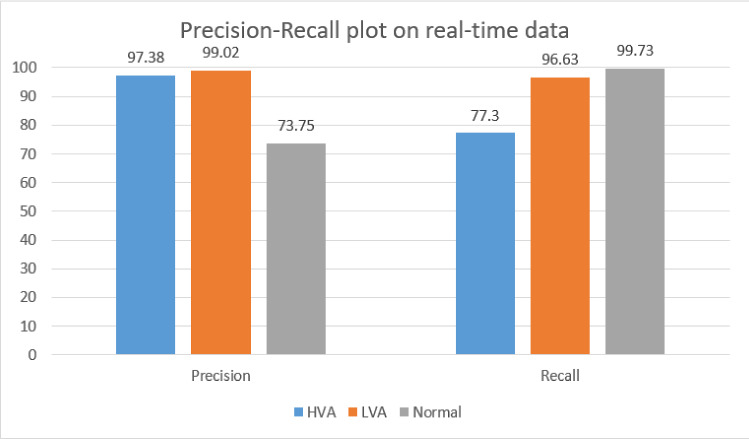
Precision recall comparison for multi-class classification

**Fig. 17:**
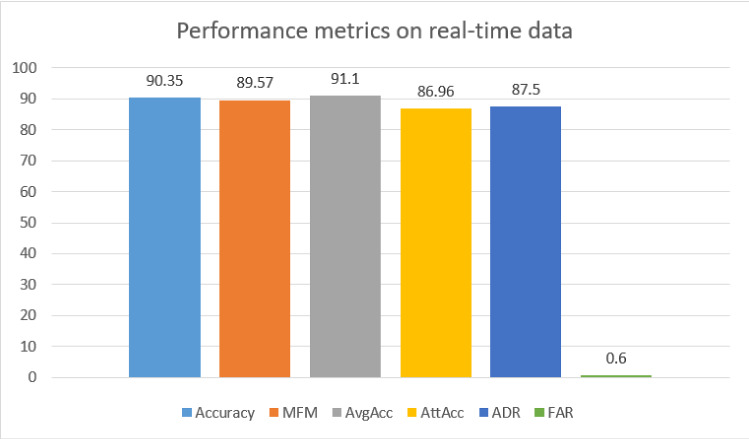
Performance matrices for multiclass classification on real-time data

Table 8 shows the confusion matrix corresponding to the multi-class classification. The precision and recall under multi-class classification are shown in Fig. 16, and performance metrics generated for the corresponding matrix are shown in Fig. 17.

It is observed that the multi-class performance has slightly decremented compared to binary class in terms of accuracy. The reason is that, in the case of multi-class classification, the prediction is more specific to each class. But, in binary class classification, all attack classes are predicted under the single aggregated attack class. E.g., assume that the misclassification of prediction in Table 8 occurred between HVA and LVA attacks i.e., 78 instances of HVA predicted as LVA and 166 instances of LVA predicted as HVA. But this misclassification is absent in the binary classification because HVA and LVA are merged into a single class.

**Table 8:** Confusion matrix for real-time test data set under multi-class classification

	HVA	LVA	Normal
HVA	6960	78	1962
LVA	166	9663	171
Normal	21	17	5994

**Fig. 18:**
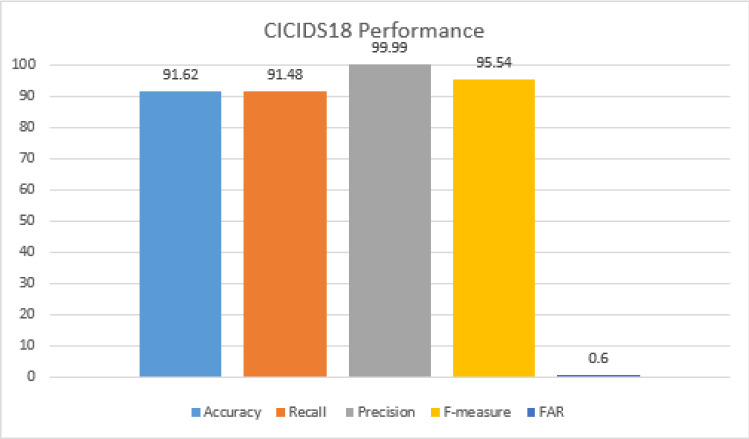
Performance matrices for binary-class classification on CICIDS18 data

**Fig. 19:**
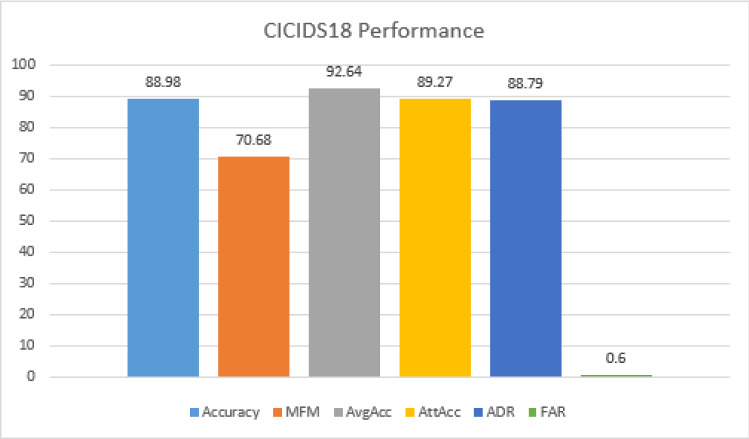
Performance matrices for multiclass classification on CICIDS18 data

*On CICIDS18 Dataset:* Confusion matrix for the binary and multi-class classification is given by Table 9 and 10. Figure 18 and 19 show the accuracy for binary and multi-class classification using the confusion matrix described above shows 91.62% and 88.98% respectively on the CICIDS18 data set. A lower precision against the normal category in multi-class classification is due to the lower number of normal packets considered for analysis against the attack packets. Hence, for the number of other categories, the packets predicted as normal category dominates the true prediction of normal packets. Figures 18 and 19 show the performance on CICIDS18 benchmark data for binary and multi-class classification.

**Table 9:** Confusion matrix for CICIDS18 under binary-class classification

Classes	Attack	Normal
Attack	314018	29243
Normal	38	5994

**Table 10:** Confusion matrix for CICIDS18 data set under multi-class classification

	HVA	LVA	Normal
HVA	242014	8054	23537
LVA	1183	62764	5706
Normal	21	17	5994

**Fig. 20:**
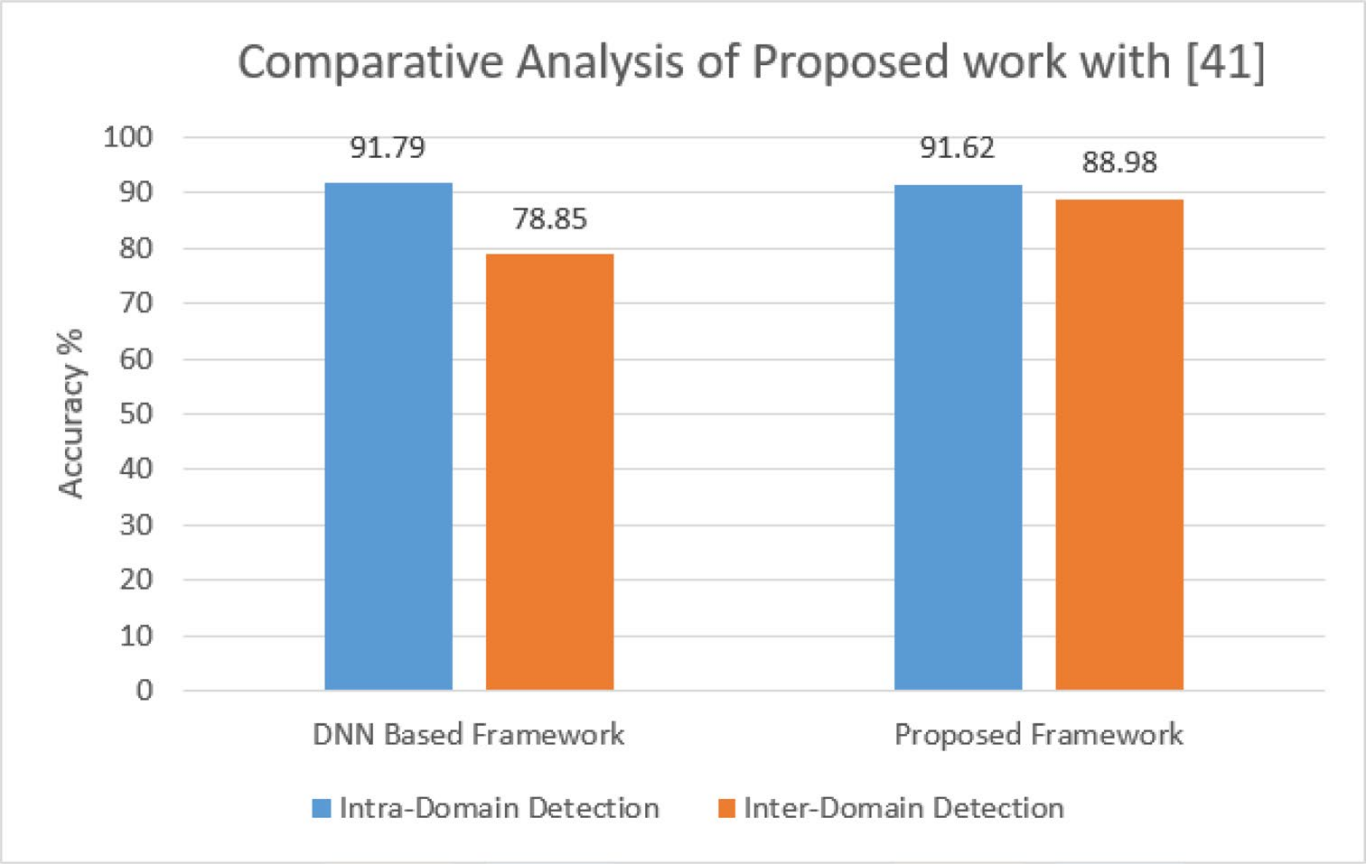
Comparative performance analysis of proposed framework with DNN-based approach [41]

*Comparison of performance with existing state-of-the-art:* The framework provided by Sameera and Shashi [41] used DNN to detect ZAs. Before detection, all the traffics first undergo soft-labeling through clustering, which further gives the facility to make the detection supervised. They use labeled attack and normal data along with the unlabeled data whose label is generated using clustering. In Fig. 20, the accuracy of model [41] and proposed approach are compared for both intra-domain and inter-domain detection. The intra-domain detection of [41] is 91.71%, which is the average attack detection accuracy of DoS-to-probe and DoS-to-R2L of the NSL-KDD data set. In the proposed approach, intra-domain detection accuracy is 91.62% for binary class classification on real-time captured traffic. In [41], all the performance analysis is done on binary class classification. So, only binary classification of the proposed framework is considered for comparative analysis.

On the other hand, in [41] inter-domain detection accuracy is 78.85% where the model is trained on the NSL-KDD data set and tested on the CIDD data set. In our approach, inter-domain detection accuracy is 88.98% where the model is trained and signature is generated on real-time captured traffic and tested on the CICIDS18 data set. So, comparing the performance of inter-domain of both models, the proposed model slightly shows an improvement in the accuracy. Unlike [41], the proposed work does not require any feature and labeling process.

## Conclusion

This paper proposes a novel robust intelligent approach to detect the signatures of ZAs. The proposed work is divided into two modules (*a*) HVA to derive high volume ZAs using heavy-hitter and (*b*) LVA to derive signatures for low volume ZAs using graph technique. The data is captured by setting up a virtual environment that consists of 10 genuine, 10 high volume attack nodes and 3 low volume attack nodes. The proposed approach works on the raw hexadecimal byte format and successfully captures unknown attacks. The result analysis of the proposed work is done for binary and multi-class classification. The binary classification performance for real-time attack data shows an accuracy of 91.33% and 90.35% for binary and multi-class classification. On the other hand, for the CICIDS18 benchmark data set, binary and multi-class classification shows the accuracy of 91.62% and 88.98%, respectively.

Several recent works are restricted to high volume ZAs [36, 59] or use an anomaly-based approach discussed in Sect. 3. This paper designs a model which detects not only high volume ZAs but also zero-day LVAs. This approach is independent of source/destination-specific information like IP address, port, etc., and can be configured to cover broader variants of ZAs.

*Limitations and Future Work of the Proposed Framework:* In the future, we would like to improve the robustness of our approach by detecting those types of ZAs whose behaviors are independent of existing attacks. We will perform tests to improve accuracy in the case of multi-class classification. We will optimize the time complexity of LVA signature generation and scan for the intrusive pattern. The limitation of the proposed work is that the exact category of LVA and HVA attack variants is not detected due to its implementation approach which will be explored in the future too.
